# Adaptive sampling dual terahertz comb spectroscopy using dual free-running femtosecond lasers

**DOI:** 10.1038/srep10786

**Published:** 2015-06-02

**Authors:** Takeshi Yasui, Ryuji Ichikawa, Yi-Da Hsieh, Kenta Hayashi, Harsono Cahyadi, Francis Hindle, Yoshiyuki Sakaguchi, Tetsuo Iwata, Yasuhiro Mizutani, Hirotsugu Yamamoto, Kaoru Minoshima, Hajime Inaba

**Affiliations:** 1 Institute of Technology and Science, Tokushima University, 2-1 Minami-Josanjima, Tokushima 770-8506, Japan; 2 Graduate School of Engineering Science, Osaka University, 1-3 Machikaneyama, Toyonaka, Osaka 560-8531, Japan; 3 JST, ERATO, MINOSHIMA Intelligent Optical Synthesizer Project, 2-1 Minami-Josanjima, Tokushima 770-8506, Japan; 4 Laboratoire de Physico-Chimie de l’Atmosphère, Université du Littoral Côte d’Opale, 189A Av. Maurice Schumann, Dunkerque 59140, France; 5 Center for Optical Research and Education, Utsunomiya University, 7-1-2, Yoto, Utsunomiya, Tochigi 321-858, Japan; 6 Graduate School of Informatics and Engineering, The University of Electro-Communications, 1-5-1, Chofugaoka, Chofu, Tokyo 182-8585, Japan; 7 National Metrology Institute of Japan, National Institute of Advanced Industrial Science and Technology, 1-1-1 Umezono, Tsukuba, Ibaraki 305-8563, Japan

## Abstract

Terahertz (THz) dual comb spectroscopy (DCS) is a promising method for high-accuracy, high-resolution, broadband THz spectroscopy because the mode-resolved THz comb spectrum includes both broadband THz radiation and narrow-line CW-THz radiation characteristics. In addition, all frequency modes of a THz comb can be phase-locked to a microwave frequency standard, providing excellent traceability. However, the need for stabilization of dual femtosecond lasers has often hindered its wide use. To overcome this limitation, here we have demonstrated adaptive-sampling THz-DCS, allowing the use of free-running femtosecond lasers. To correct the fluctuation of the time and frequency scales caused by the laser timing jitter, an adaptive sampling clock is generated by dual THz-comb-referenced spectrum analysers and is used for a timing clock signal in a data acquisition board. The results not only indicated the successful implementation of THz-DCS with free-running lasers but also showed that this configuration outperforms standard THz-DCS with stabilized lasers due to the slight jitter remained in the stabilized lasers.

Terahertz (THz) radiation, lying in the spectral region between 0.1 and 10 THz, has attracted much attention as a new tool for material characterization and sensing because it allows us to observe interesting phenomena that are characteristic to this region, such as optical phonon scattering and plasma frequency in solids, dielectric properties (including ionic polarization and orientation polarization), rotational transitions of polar gas molecules, and intermolecular interactions^1^. Therefore, the development of spectroscopic analysis has been a principal driving force in developing THz technology. To probe a variety of phenomena in the THz region and characterise them precisely, a spectroscopic technique with high accuracy, high resolution, and wide spectral coverage is strongly desired. Unfortunately, all of these requirements cannot be satisfied at the same time using standard THz spectroscopic techniques, including THz time-domain spectroscopy (THz-TDS) with coherent broadband THz radiation^2^, far-infrared Fourier transform spectroscopy with incoherent broadband THz radiation^3^, and THz frequency-domain spectroscopy (THz-FDS) with coherent tunable continuous-wave THz (CW-THz) radiation^4^.

Recently, the development of THz frequency combs has unlocked further potential for high-accuracy, high-resolution, broadband THz spectroscopy^5^,^6^,^7^. THz spectroscopy based on a THz comb can combine the advantages of THz-TDS and THz-FDS, namely, broadband coverage and high resolution, because the mode-resolved THz comb spectrum includes both broadband THz radiation and narrow-line CW-THz radiation characteristics. Furthermore, absolute accuracy of all frequency modes in a THz comb is ensured by phase-locking of the THz comb to a microwave frequency standard via laser control. The mode-resolved THz comb spectrum can be obtained by dual comb spectroscopy (DCS) techniques in the time^7^,^8^,^9^,^10^,^11^ and frequency domains^5^,^6^. The spectrum obtained by THz-DCS is composed of a series of frequency spikes, separated by the mode-locked frequency, with a linewidth equal to the reciprocal of the observation window duration of the temporal waveform. Therefore, each comb mode can be used as a universal frequency scale for broadband THz radiation. Furthermore, the combination of the spectral interleaving technique^12^,^13^ with DCS has yielded a gapless mode-resolved comb spectrum, with considerable enhancements in spectral resolution and accuracy^14^. These previous THz-DCS techniques require precisely stabilized, dual femtosecond lasers, which has often restricted the use of THz-DCS in various applications, despite its superior spectroscopic performance. If THz-DCS could be implemented using *free-running*, that is, *unstabilized*, lasers, the scope of applications of the THz-DCS would be greatly expanded.

More recently, the adaptive sampling technique has been proposed for DCS in the visible^15^ and near-infrared regions^16^, allowing the use of dual free-running lasers by correcting the fluctuation of the time axis using an adaptive sampling clock. However, to date there have been no attempts to apply the adaptive sampling technique to THz-DCS. In this article, we report THz-DCS with dual free-running lasers by modifying the adaptive sampling technique to work with a THz comb. THz-comb-referenced spectrum analysers^17^,^18^,^19^,^20^,^21^ have been constructed with a dual configuration related to dual free-running lasers, and these were effectively used in our system to generate an adaptive sampling clock for THz-DCS.

## Principle

**Figure 1:**
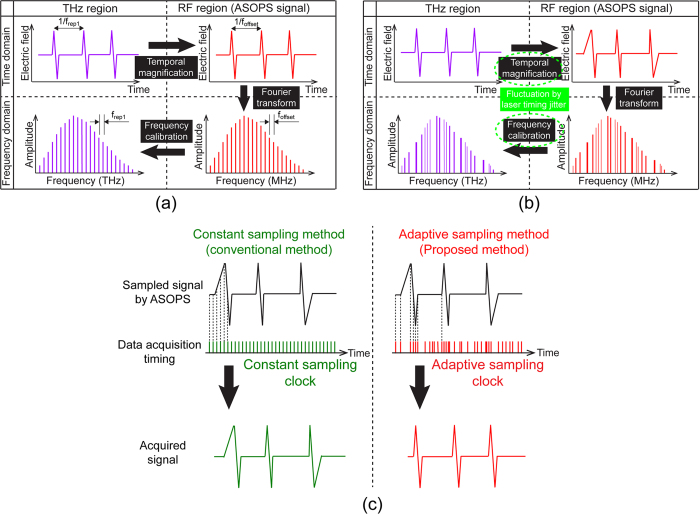
Principle of operation. Signal chart of THz-DCS in THz and RF regions (**a**) when the influence of the laser timing jitter is negligible and (**b**) when the timing jitter fluctuates *TMF*. (**c**) Comparison of the data acquisition between the constant sampling method and the adaptive sampling method.

Since principle of the adaptive sampling method is given in detail elsewhere^15^,^16^, we here explain this method from the viewpoint of THz-DCS. Figure 1(a) shows a signal flowchart of THz-DCS in THz and radio-frequency (RF) regions when the influence of the laser timing jitter is negligible. The temporal waveform of a THz pulse train is acquired over many repetition periods using asynchronous optical sampling (ASOPS) with two mode-locked femtosecond lasers (repetition frequencies = *f*_*rep1*_ and *f*_*rep2*_) with a frequency offset between them (=*f*_*offset*_ = *f*_*rep2*_ - *f*_*rep1*_)^22^,^23^,^24^,^25^. As a result of using ASOPS, the repetition period of the THz pulse train (=*1/f*_*rep1*_) is expanded to *1/f*_*offset*_, based on a temporal magnification factor (*TMF*) of *f*_*rep1*_*/f*_*offset*_. The temporal signal slowed-down to the RF region, namely, an RF pulse train, can be directly measured in a data acquisition board of a computer without the need for mechanical time-delay scanning. The mode-resolved comb spectrum is obtained in the RF region, namely, an RF comb, by calculating the Fourier transform (FT) of the RF pulse train. Finally, the THz comb can be obtained by calibrating the frequency scale of the RF comb with the *TMF*. If we use dual free-running lasers in ASOPS, the timing jitter between them results in significant variations in *TMF*, constantly fluctuating the time axis of the RF pulse train as shown in Fig. 1(b). Such fluctuation of the time scale propagates to the frequency scales of the RF comb and the THz comb via the FT and frequency calibration, leading to severe degradation of the spectral resolution and accuracy.

When the temporal waveform of this distorted RF pulse train is acquired in synchronization with a constant sampling clock using the data acquisition board (in other words, the constant sampling method), the time scale of the acquired signal remains distorted, as shown in the left part of Fig. 1(c). However, if the temporal waveform of the distorted RF pulse train is acquired in synchronization with an adaptive sampling clock (in other words, the adaptive sampling method), reflecting the rapid fluctuations of the *TMF* caused by the timing jitter, the fluctuation of the time scale in the RF pulse train can be cancelled, as shown in the right part of Fig. 1(b).

**Figure 2:**
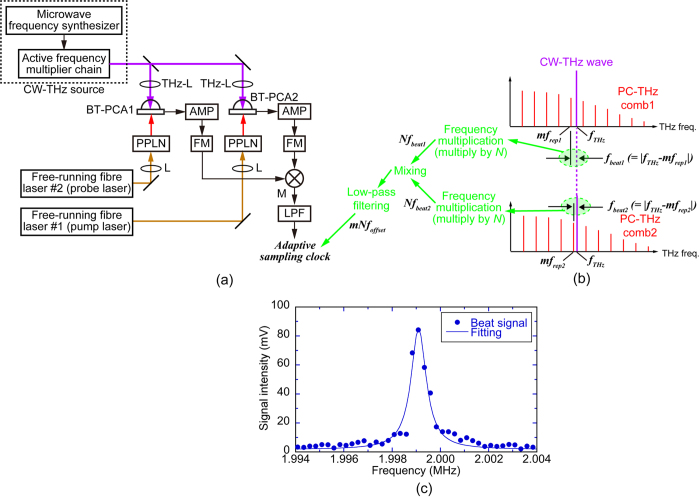
(**a**) Experimental setup of adaptive-sampling-clock generator based on dual THz-comb-referenced spectrum analysers . BT-PCA1 and BT-PCA2, bowtie-shaped, low-temperature-grown, GaAs photoconductive antennas; Ls, objective lenses; PPLNs, periodically-poled-lithium-niobate crystals; THz-Ls, THz Teflon lenses; AMP, current preamplifier; FMs, frequency multipliers (frequency multiplication factor =*N *= 40); M, double-balanced mixer; LPF, low-pass filter. (**b**) Extraction of beat signal between dual THz comb modes based on dual THz-comb-referenced spectrum analysers. (**c**) Extracted beat signal between dual THz comb modes around 4 THz.

Here we consider how we can extract an adaptive sampling clock signal sensitively reflecting the timing jitter between free-running lasers. It is essential that variations in both *f*_*rep1*_ and *f*_*rep2*_ should be simultaneously accounted for because THz comb is a harmonic comb of the repetition frequency without any frequency offset. The most elegant way to do this is to directly measure *f*_*offset*_, which contains all the required information in a single measured parameter. Therefore, the adaptive sampling clock for THz-DCS should be derived from *f*_*offset*_ with the high-order harmonic components offering a greater degree of sensitivity. The dual THz combs contain this information via access to high-order harmonics of *f*_*rep1*_ and *f*_*rep2*_. To this end, two beat signals were extracted from the dual THz comb modes by modifying and extending a THz-comb-referenced spectrum analyzer^17^,^18^ into a dual configuration. Figures 2(a,b) illustrate the experimental setup of this method and the corresponding spectral behavior. When dual free-running mode-locked Er-doped fibre laser beams at a wavelength of 1.5 μm (*f*_*rep1*_ ≈ 100 MHz, *f*_*rep2*_ ≈ 100 MHz + 50 Hz, and *f*_*offset*_ ≈ 50 Hz) are individually focused on bowtie-shaped, low-temperature-grown, GaAs photoconductive antennas (BT-PCA1 and BT-PCA2) after the wavelength conversion by second-harmonic-generation (SHG) with periodically-poled-lithium-niobate (PPLN) crystals. The photocarrier THz combs with a frequency spacing of *f*_*rep1*_ and *f*_*rep2*_ (PC-THz combs 1 and 2) are respectively induced in BT-PCA1 and BT-PCA2. A CW-THz wave (output frequency =*f*_*THz*_ = 0.1 THz, linewidth < 0.6 Hz, average power =2.5 mW) from a combination of an active frequency multiplier chain with a microwave frequency synthesizer is also incident on both BT-PCA1 and BT-PCA2. As a result of photoconductive mixing in BT-PCA1 and BT-PCA2, a pair of beat signals between the CW-THz wave and PC-THz combs 1 and 2 is generated in the RF region. If the order of the PC-THz comb modes *m* is nearest in frequency to the CW-THz wave (=1,000), then the two beat signals (*f*_*beat1*_ and *f*_*beat2*_) can be expressed as |*f*_*THz*_ − *mf*_*rep1*_| and |*f*_*THz*_ *– mf*_*rep2*_|, respectively. The beat signals are frequency multiplied by frequency multipliers (FM, frequency multiplication factor = *N* = 40) before being electrically mixed together by a double-balanced mixer (M) and filtered by a low-pass filter (LPF). The resulting signal represents the beat signal between the dual THz comb modes at 4 THz (freq. = *mNf*_*offset*_). Figure 2(c) shows the spectrum of the extracted signal, which has the nominal desired clock frequency (=1.999 MHz) with a narrow linewidth (=800 Hz) so that it can be used for the adaptive sampling clock in THz-DCS. It is important to note that only a single adaptive sampling clock is required for THz-DCS because the THz comb is a harmonic frequency comb of *f*_*rep1*_ with no carrier-envelope-offset frequency (*f*_*ceo*_). The simplicity of generating the adaptive sampling clock is advantageous compared with adaptive sampling DCS in the visible and near-infrared regions, where two kinds of adaptive sampling clocks are required to correct variations of both *f*_*rep1*_ and *f*_*ceo*_^15^,^16^.

## Results

**Figure 3:**
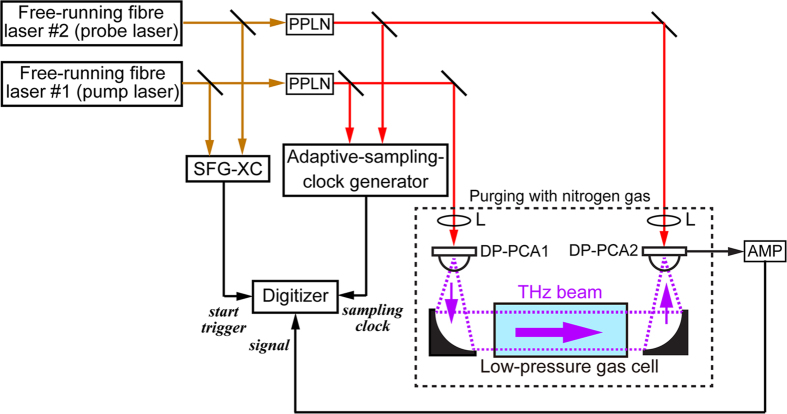
Experimental setup of adaptive sampling THz-DCS. SFG-XC, sum-frequency-generation cross-correlator; PPLNs, periodically-poled-lithium-niobate crystals; Ls, objective lenses; DP-PCA1, dipole-shaped, low-temperature-grown, GaAs photoconductive antenna for THz emitter; DP-PCA2, dipole-shaped, low-temperature-grown, GaAs photoconductive antenna for THz detector; AMP, current preamplifier.

Figure 3 is a schematic diagram of the experimental setup, which contained dual free-running mode-locked Er-doped fibre lasers at a wavelength of 1.5 μm (*f*_*rep1*_ ≈ 100 MHz, *f*_*rep2*_ ≈ 100 MHz + 50 Hz, and *f*_*offset*_ ≈ 50 Hz), a sum-frequency-generation cross-correlator (SFG-XC) for the start trigger signal, an adaptive-sampling-clock generator for timing clock signal, a THz optical setup containing a low-pressure gas cell, and the data acquisition. The temporal waveform of the RF pulse train (repetition period =1/*f*_*offset*_ ≈ 20 ms) was acquired within a time window size of 200 ms with a digitizer (sampling rate = 2 × 10^6^ samples/s, number of sampling points = 400,000, resolution = 20 bit) by using the adaptive sampling clock as a timing signal of the data acquisition in the digitizer. This configuration with a *TMF* around 2,000,000 (=*f*_*rep1*_/*f*_*offset*_) is equivalent to the acquisition of 10 consecutive THz pulses (repetition period =1/*f*_*rep1*_ ≈ 10 ns), corresponding to a sampling interval of 100 fs with a time window of 100 ns. We acquired temporal waveforms of the pulse train at a scan rate of 5 Hz and accumulated them to optimise the signal-to-noise ratio (SNR) and dynamic range (DR). Finally, the mode-resolved THz comb spectrum was obtained by FT and frequency calibration.

**Figure 4:**
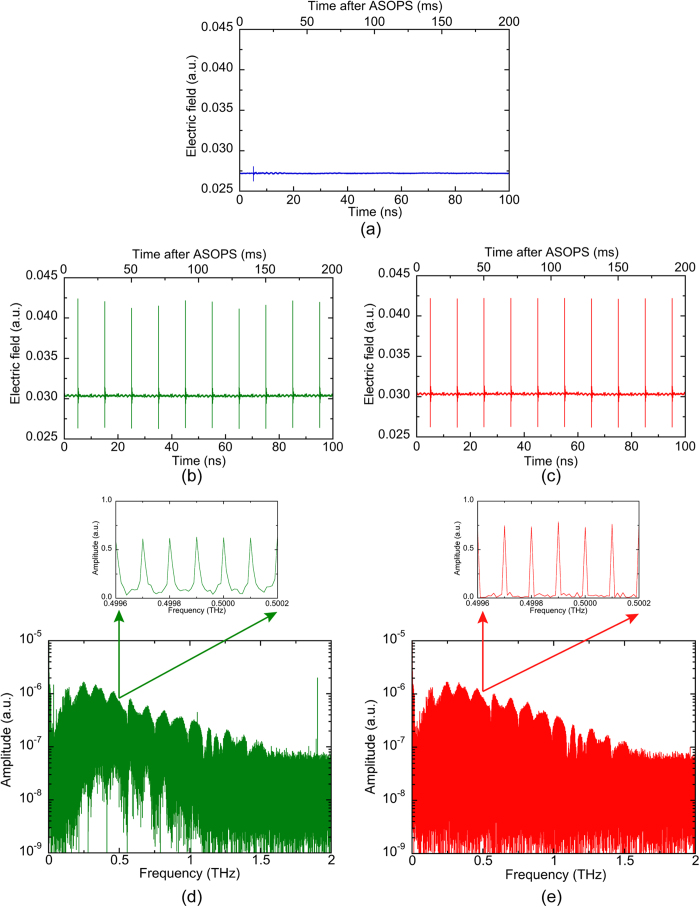
Comparison of the accumulated temporal waveforms (accumulation number = 10,000) obtained with (**a**) the constant sampling method with free-running lasers, (**b**) the constant sampling method with stabilized lasers, and (**c**) the adaptive sampling method with free-running lasers. Comparison of the mode-resolved THz comb spectrum between (**d**) constant sampling method with stabilized lasers obtained by FT of Fig. 4(b) adaptive sampling method with free-running lasers obtained by FT of Fig. 4(c).

To investigate the effectiveness of the adaptive sampling method in the time domain, we accumulated 10,000 temporal waveforms, each of 10 consecutive THz pulses, and compared the results obtained with the constant sampling method and the adaptive sampling method, as shown in Fig. 4. The accumulated temporal waveform is shown in the three upper panes for: (a) the constant sampling method with free-running lasers; (b) the constant sampling method with stabilized lasers [RMS timing jitter (0.1 Hz – 500 kHz) < 150 fs]; and (c) the adaptive sampling method with free-running lasers. The upper horizontal scales indicate the time scale after ASOPS whereas the lower horizontal scales indicate real time scale calibrated by *TMF*. In Fig. 4(a), the signal of the THz pulse train was almost unobservable except for the first THz pulse. This is caused by the instability of the *TMF*, which changes the temporal positions of the 10 consecutive THz pulses in each data acquisition. The accumulation of the waveforms results in the signal being buried in the noise. For this reason, the constant sampling method cannot be used with free-running lasers. In contrast, as shown in Fig. 4(b), 10 consecutive THz pulses were observed even after the signal accumulation, which is similar to previous studies employing the constant sampling method with stabilized lasers^7^. However, the peak amplitude of the pulsed THz electric field varied with each pulse. This result implies that the remaining timing jitter in dual stabilized femtosecond lasers caused imperceptible fluctuations in the relative timing between the THz pulse and the probe pulse even though *f*_*rep1*_ and *f*_*rep2*_ were stabilized by the control system. As shown in Fig. 4(c), despite the free-running operation of the dual lasers, the 10 consecutive THz pulses were clearly observed in the accumulated temporal waveform. Furthermore, it should be emphasized that the peak amplitude of the THz electric field was constant for all pulses, in contrast to Fig. 4(b). That is to say, the adaptive sampling method seems to be more powerful than the constant sampling method with stabilized lasers, which is limited by the residual timing jitter.

To investigate the effectiveness of the adaptive sampling method in the frequency domain, we obtained a mode-resolved THz comb spectrum by calculating the FT of the temporal waveform in Fig. 4(b,c). Figure 4(d,e) show a comparison of the mode-resolved THz comb spectra between the constant sampling method with stabilized lasers and the adaptive sampling method with free-running lasers. The spectral envelopes of these two THz combs were identical to each other, revealing the periodic modulation due to the multiple reflections in photoconductive antennas used in THz-DCS setup and the sharp spectral dips caused by atmospheric water vapor. However, the noise floor in the THz comb spectrum swelled in Fig. 4(d). To observe the detailed mode structure of the THz comb, we expanded the spectral region around 0.5 THz. As shown in the insets of Fig. 4(d,e), the THz comb modes had a frequency spacing of 100 MHz. However, the linewidth of the comb mode in Fig. 4(d) (=17 MHz) was larger than that in Fig. 4(e) (=10 MHz), once again due to the residual jitter of the stabilized lasers. Furthermore, the amplitude contrast between the comb mode and gaps is higher when the adaptive sampling method is used rather than the constant sampling method. Specifically, the comb modes in Fig. 4(d) and its inset exhibited a poorer DR compared with those in Fig. 4(e) and its inset, which maintain sharper distinct spikes. The increased linewidth and lower contrast obtained by the constant sampling method lead to degraded spectral resolution and DR. Hence, the adaptive sampling method not only enables use of free-running lasers but also has the potential to enhance the spectroscopic performance over that of conventional THz-DCS based on the constant sampling method with stabilized lasers due to the slight jitter remained in the stabilized lasers.

**Figure 5:**
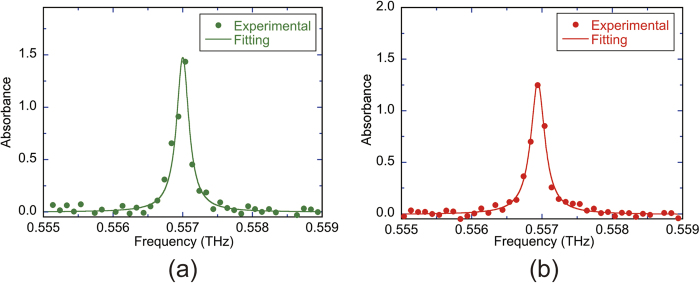
Comparison of absorbance spectrum of the water rotational-transition (1_10_ ← 1_01_) between (**a**) the constant sampling method with stabilized lasers and (**b**) the adaptive sampling method with free-running lasers.

THz spectroscopic analysis of low-pressure molecular gas is one of the interesting applications that require the highest resolution and accuracy in the broadband THz spectral range. We evaluated the potential of the proposed system for high-accuracy, high-resolution, broadband THz spectroscopy by measuring low-pressure molecular gasses. The performance was first assessed for an isolated, sharp absorption line, and the absorption spectrum of the rotational transition 1_10_ ← 1_01_ of water vapor (freq. =0.556936 THz) was measured. A mixture of water vapour (partial pressure = 145 Pa) and nitrogen (partial pressure =2555 Pa) was introduced into a low-pressure gas cell. The pressure broadening linewidth of this absorption line was estimated to be 201 MHz from the self and collision pressure-broadening coefficients^26^. Figure 5(a,b) show a comparison of the absorbance spectrum between the constant sampling method with stabilized lasers and the adaptive sampling method with free-running lasers. By fitting the measured spectral profile with a Lorentzian function, the center frequency and the spectral linewidth of the absorption line were determined to be 0.557003 THz and 238 MHz for Fig. 5(a) and 0.556935 THz and 243 MHz for Fig. 5(b), respectively. The deviations of the determined center frequency from the spectral database values^27^ were 67 MHz for Fig. 5(a) and 1 MHz for Fig. 5(b), which were both within the comb-mode frequency spacing of 100 MHz. On the other hand, the determined linewidths were in reasonable agreement with the expected pressure-broadening linewidth. This comparison indicated that adaptive sampling THz-DCS with free-running lasers could achieve a spectroscopic performance similar to or greater than that of constant sampling THz-DCS with stabilized lasers for high-precision spectroscopy of low-pressure gases.

**Figure 6:**
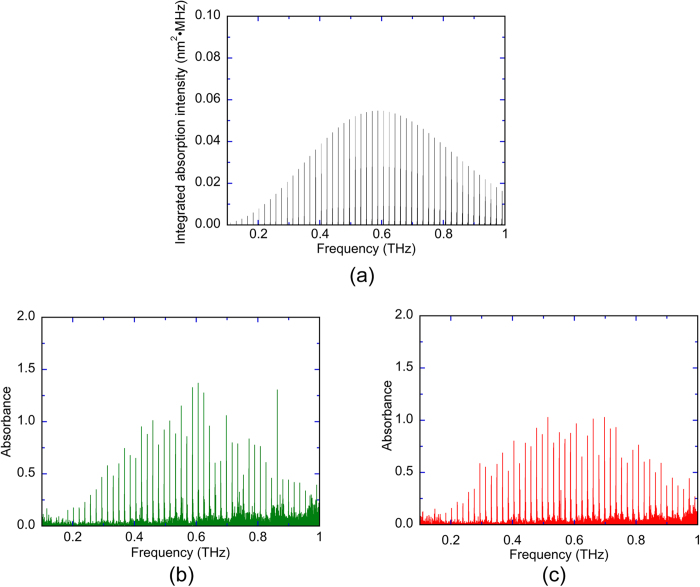
Comparison of absorption/absorbance spectrum of low-pressure CH_3_CN gas obtained (**a**) from the tabulated data^28^, (**b**) using the constant sampling method with stabilized lasers, and (**c**) using the adaptive sampling method with free-running lasers.

Finally, to demonstrate the capacity to simultaneously probe multiple absorption lines of low-pressure molecular gas, we performed gas-phase spectroscopy of acetonitrile (CH_3_CN). Since CH_3_CN is not only a very abundant species in the interstellar medium but also one of the volatile organic compounds present in the atmosphere, its spectroscopic analysis is important in astronomy and atmospheric pollution. As CH_3_CN has a symmetric top molecular structure with a rotational constant*, B*, of 9.2 GHz, many groups of rotational transitions regularly spaced by *2B* (=18.4 GHz) appear in the THz region, as shown by the tabulated data in Fig. 6(a)^28^. Figure 6(b,c) show a comparison of the absorbance spectrum of CH_3_CN at 1 kPa between the constant sampling method with stabilized lasers and the adaptive sampling method with free-running lasers, respectively. The groups of rotational transitions regularly spaced by 18.4 GHz (=*2B*) were clearly confirmed within a frequency range of 1 THz in Fig. 6(b,c), allowing 45 groups to be assigned (from J = 9 around 0.18 THz to J = 53 around 0.98 THz), indicating the capacity to probe multiple absorption lines simultaneously. The spectral envelope of absorbance peaks for both were uneven and not smooth compared with Fig. 6(a). One reason for such unevenness is spectral mismatching between discretely mode-resolved THz comb spectrum and sharp absorption lines into each rotational-transitions group, which does not always indicate the limit of the spectroscopic performance in those methods. However, Fig. 6(b) was more uneven than Fig. 6(c) due to increase of noise level. This implies that the adaptive sampling method improves the SNR. From these results, we can conclude that adaptive sampling THz-DCS has great potential for high-accuracy, high-resolution, broadband THz spectroscopy and is optimally employed with free-running lasers.

## Discussion

**Figure 7:**
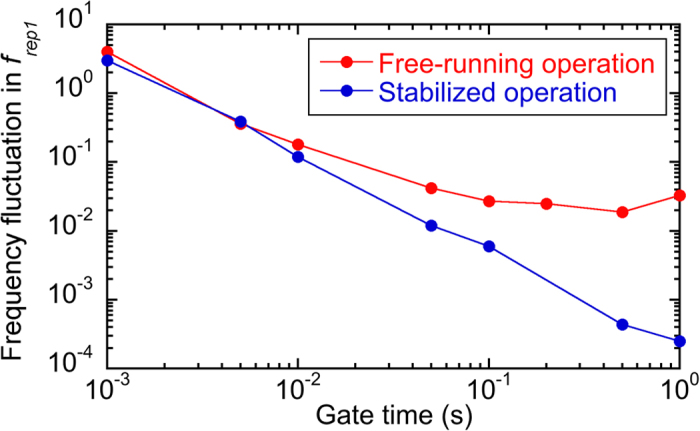
Comparison of frequency fluctuation in *f_rep1_* between free-running and stabilized femtosecond lasers with respect to gate time.

In the proposed method, the fluctuation of the frequency axis in the mode-resolved THz comb spectrum was effectively cancelled by the data acquisition in synchronization with the adaptive sampling clock. Nevertheless, the absolute frequency accuracy of the spectrum was not ensured because *f*_*rep1*_ may display rapid fluctuations due to the free-running laser operation. The frequency fluctuation of *f*_*rep1*_ with respect to gate time in the free-running laser is shown as red plots in Fig. 7. The variation of *f*_*rep1*_ in the free-running laser was measured to be 25 mHz over 200 ms, corresponding to a single scan time for one temporal waveform of 10 consecutive THz pulses (=10/f_offset_). Since this frequency fluctuation is multiplied by a ratio of frequency spacing to THz comb-mode frequency (=1 THz/100 MHz = 10,000), the mode-resolved THz comb spectrum includes a fluctuation of 250 Hz at 1 THz, corresponding to a frequency accuracy of 2.5 × 10^−10^. Since this fluctuation is much smaller than frequency spacing and linewidth of the comb modes, it remains negligible in THz-DCS.

The spectral resolution in DCS is determined by the linewidth of the comb mode because the linearity of the frequency scale is recovered by using the adaptive sampling method. The comb-mode linewidth of 10 MHz observed in the inset of Fig. 4(e) was dependent on the time window size of 100 ns in Fig. 4(c). If the time window size is further increased, the linewidth is reduced. However, the intrinsic limit is the relative linewidth between dual THz comb modes. An estimation of this linewidth is provided by 200 Hz at 1 THz from the linewidth (=800 Hz) of the beat signal between dual THz comb modes at 4 THz presented in Fig. 2(c). Therefore, the present system is capable of achieving further linewidth improvements if the time window size is further expanded.

In the proposed approach, we used dual free-running femtosecond lasers having very close repetition frequencies, namely low *f*_*offset*_. One may consider the limit of applicability of this approach to dual lasers with significantly different repetition frequencies. The *f*_*offset*_ value around 50 Hz was selected by considering *f*_*rep1*_ (≈100 MHz) and the frequency bandwidth of a current preamplifier (=1 MHz) connected with DP-PCA2 because the required frequency bandwidth was determined by *TMF* (=*f*_*rep1*_/*f*_*offset*_). If dual femtosecond lasers with higher *f*_*rep1*_ and *f*_*rep2*_ are used together with faster free-space electro-optics sampling (FSEOS) detection, *f*_*offset*_ will be increased up to several kHz. For example, *f*_*offset*_ of 2 kHz has been achieved in ASOPS-THz-TDS where *f*_*rep1*_ ≈ *f*_*rep2*_ ≈ 1 GHz and the frequency bandwidth in the FSEOS detection was 100 MHz^24^. For further larger *f*_*offset*_, the adaptive sampling method may be combined with arbitrary detuning ASOPS^29^ or DCS with quasi-integer-ratio repetition rates^30^.

## Conclusions

To promote the wide use of THz-DCS in THz spectroscopic applications, we developed an adaptive sampling method for THz-DCS with free-running femtosecond lasers. A narrow-linewidth beat signal between dual THz comb modes at 4 THz was extracted by using dual THz-comb-referenced THz spectrum analysers. The derived electrical signal was used as a high-quality adaptive sampling clock for the data acquisition of THz-DCS. As a result, the fluctuation in the time scale, induced by the laser timing jitter, was fully corrected, and distinct comb modes were achieved in the mode-resolved THz comb spectrum. The demonstrated results show successful implementation of THz-DCS with free-running lasers. More interestingly, their results revealed the superior spectroscopic performance of this method over conventional constant sampling THz-DCS with stabilized lasers due to the slight jitter remained in the stabilized lasers. Although it has been demonstrated that the adaptive sampling DCS with free-running lasers is superior to the constant sampling DCS with free-running lasers^15^,^16^, it is the first time to demonstrate the superiority of this method over the constant sampling DCS with commercially available, stabilized lasers to the best of our knowledge.

In this article, the discrete spectral distribution of the THz comb mode limits the spectral sampling interval to *f*_*rep1*_ rather than the comb mode linewidth. However the combination of adaptive sampling THz-DCS with the spectral interleaving technique^14^ will reduce the spectral sampling interval to the comb-mode linewidth. In this case, the potential of the distinct, narrow linewidth comb modes [see the inset of Fig. 4(e)] will be fully harnessed. Furthermore, the application of the adaptive sampling method will enable ASOPS-THz-TDS^22^,^23^,^24^,^25^ with free-running lasers, which is more widely employed for THz spectroscopy rather than high-precision THz-DCS. The proposed method will lower the barriers for their practical use, and will hence accelerate their real world applications.

## Methods

### Experimental setup of THz-DCS

We used dual mode-locked Er-doped fibre lasers (ASOPS TWIN 100 with P100, Menlo Systems; centre wavelength *λ*_*c *_= 1550 nm, pulse duration *∆τ* = 58 fs, mean power *P*_*mean *_= 280 mW) for adaptive sampling THz-DCS (see Fig. 3). In the case of the adaptive sampling THz-DCS with dual free-running lasers, the repetition frequencies of the lasers (*f*_*rep1*_ ≈ 100 MHz, *f*_*rep2*_ ≈ 100 MHz + 50 Hz) and the frequency offset between them (*f*_*offset*_ = *f*_*rep2*_–*f*_*rep1*_ ≈ 50 Hz) were not stabilized. On the other hand, in the case of the constant sampling THz-DCS with dual stabilized lasers, *f*_*rep1*_ and *f*_*rep2*_ were independently stabilized by two control systems [ASOPS, Menlo Systems; RMS timing jitter (0.1 Hz – 500 kHz) < 150 fs] referencing a rubidium frequency standard (output frequency = 10 MHz, accuracy = 5 × 10^−11^, stability = 2 × 10^−11^ at 1 s) as a time base (not shown in Fig. 3). Figure 7 shows a comparison of frequency fluctuation in *f*_*rep1*_ between free-running and stabilized femtosecond lasers. The difference of frequency fluctuation between them was obvious at the gate time longer than 10 ms. Portions of output beams from the two free-running lasers were used to drive an SFG-XC. The resulting SFG signal was used to generate a time origin signal in the ASOPS measurement. After wavelength conversion of the two laser beams by SHG with PPLN crystals, portion of two SHG beams (pump light and probe light) were fed into an adaptive-sampling-clock generator to generate the adaptive sampling clock in the THz region [see Fig. 2]. Then, pulsed THz radiation was emitted from a dipole-shaped, low-temperature-grown (LTG), GaAs photoconductive antenna (DP-PCA1) triggered by pump light (*λ*_*c *_= 775 nm, *∆τ* = 80 fs, *P*_*mean *_= 20 mW), passed through a low-pressure gas cell (length = 500 mm, diameter = 40 mm), and was then detected by another dipole-shaped LTG GaAs photoconductive antenna (DP-PCA2) triggered by probe light (*λ*_*c *_= 775 nm, *∆τ* = 80 fs, *P*_*mean *_= 10 mW). The optical path in which the THz beam propagated, except for the part in the gas cell, was purged with dry nitrogen gas to avoid absorption by atmospheric moisture. After amplification with a current preamplifier (AMP, bandwidth = 1 MHz, gain = 4 × 10^6^ V/A), the temporal waveform of the output current from DP-PCA2 was acquired with a digitizer (sampling rate = 2 × 10^6^ samples/s, resolution =20 bit) by using the SFG-XC’s output as a start trigger signal and the adaptive-sampling-clock generator’s output as a clock signal. Then, the time scale of the observed signal was multiplied by the nominal *TMF* (=*f*_*rep1*_*/f*_*offset*_ ≈ 2,000,000). This sampling rate and this *TMF* enabled us to measure the temporal waveform of 10 consecutive THz pulses at a sampling interval of 100 fs and with a time window size of 100 ns.

## Additional Information

**How to cite this article**: Yasui, T. *et al.* Adaptive sampling dual terahertz comb spectroscopy using dual free-running femtosecond lasers. *Sci. Rep.* **5**, 10786; doi: 10.1038/srep10786 (2015).
